# Global, regional, and national burdens of cirrhosis in children and adolescents aged under 19 years from 1990 to 2019

**DOI:** 10.1007/s12072-023-10531-y

**Published:** 2023-04-26

**Authors:** Chi Zhang, Yiqi Liu, Hong Zhao, Guiqiang Wang

**Affiliations:** 1 Department of Infectious Disease, Center for Liver Disease, Peking University First Hospital, Beijing, 100034 China; 2 Department of Infectious Diseases, Peking University International Hospital, Beijing, 102206 China

**Keywords:** Cirrhosis, Global burden of disease, Children, Adolescents, Trend, Incidence, Prevalence, Deaths, Disability-adjusted life-years

## Abstract

**Background & aims:**

Cirrhosis was the leading cause of morbidity and mortality in adults, but data on the burden and trends were sparse in children and adolescents. We aimed to assess the trends in 204 countries and territories over the past 30 years in children and adolescents aged 0–19 years.

**Methods:**

Data on cirrhosis was collected by the Global Burden of Disease (GBD) 2019 database from 1990 to 2019. We reported on the number, rates, and average annual percentage changes (AAPCs) of incidence and disability-adjusted life-years (DALYs) of cirrhosis at global, regional, and national level.

**Results:**

Globally, the incident numbers of cirrhosis in children and adolescents increased from 204,767 in 1990 to 241,364 in 2019, an increase of 17.9%, with an AAPC 0.13(0.10 to 0.16). Prevalence (AAPC = − 2.27[− 2.39 to − 2.15]), mortality (AAPC = − 1.68 [− 1.86 to − 1.5]), and DALYs rate (AAPC = − 1.72[− 1.88 to − 1.56]) of cirrhosis have decreased significantly. Cirrhosis incident rates varied between different ages. Cirrhosis caused by alcohol use (AAPC = 1[0.8 to 1.1]; incidence cases increased 48%), hepatitis C (AAPC = 0.4 [0.4 to 0.5]), NAFLD (AAPC = 0.5 [0.3 to 0.6]) have been increasing, while only hepatitis B (− 0.3[− 0.4 to − 0.2]) decreasing. Incidence cases of cirrhosis were increased in low (101.6%) and low-middle sociodemographic index (SDI 21.1%) areas, while decreasing in middle and above SDI areas. At the regional level, the largest increases count was observed in Sub-Saharan Africa.

**Conclusions:**

Global incidence rate of cirrhosis has been increasing, while the DALYs rate has been decreasing in children and adolescents. Morbidity of cirrhosis caused by hepatitis B declined, while hepatitis C, NAFLD, and alcohol use increased.

**Supplementary Information:**

The online version contains supplementary material available at 10.1007/s12072-023-10531-y.

## Introduction

Cirrhosis and chronic liver diseases (collectively referred to as cirrhosis) constituted majority of causes among liver-related death globally [1, 2]. It was estimated that cirrhosis caused more than 1.32 million deaths [2]. The causes of cirrhosis were complex and varied. The common causes of cirrhosis included hepatitis B virus (HBV) infection [3], hepatitis C virus (HCV) infection [4], alcoholic liver disease (ALD) [5], non-alcoholic fatty liver disease (NAFLD) [6] and other causes [7]. Mortality rate of cirrhosis among adolescents aged 10–24 years in the EU ranked first in digestive system diseases, which was at least three times higher than other digestive diseases [8]. An Ontario, Canada follow-up study (from 1997 to 2017) in the general pediatric population showed that the incidence of pediatric cirrhosis increased fourfold (2.7/100000 person-years in 1997 vs. 10.6/100000 person-years in 2017) over the past 2 decades [9]. Global Strategy of “countdown to 2030: tracking progress towards universal coverage for reproductive, maternal, newborn, and child health” has reinforced the importance of tracking children health [10]. However, children and adolescents remained a neglected age group in the quest for universal health coverage [11]. Cirrhosis prevalence or death estimates for adults have been widely published; however, the disease burden in the pediatric population remained poorly documented and understood [12].

This study aimed to investigate the global, regional, and national burden of cirrhosis in children and adolescents from 1990 to 2019. We examined the global burden (incidence, disability-adjusted life years (DALYs), prevalence, and mortality) of cirrhosis using estimates from the Global Burden of Diseases, Injuries, and Risk Factors Study (GBD) 2019 [1, 13]. Then, we stratified the trends by causes, age, sex, and sociodemographic index (SDI) groups. We also reported trends between SDI and incidence, DALYs, prevalence, and mortality.

## Methods

### Data sources

GBD 2019 provides the most up-to-date estimation of the epidemiological data of 369 diseases by age, sex, year, and geographical location from 1990 to 2019. Annual incidence cases and rate (per 100,000 population), prevalent cases and rate (per 100,000 population), death cases and rate (per 100,000 population), DALYs counts and rate (per 100,000 person-year) data, by sex, age, etiology, region and country, were collected from the Global Health Data Exchange (GHDx) query tool [14]. To estimate the burden of diseases and injuries, the GBD process involves identifying various data sources that are relevant to each condition. These sources include censuses, household surveys, civil registration and vital statistics, disease registries, health service usage data, air pollution monitors, satellite imaging, disease notifications, and other relevant sources. The identification of these data sources is carried out through a comprehensive assessment of published studies, perusal of government and international organization websites, analysis of published reports, primary data sources like the Demographic and Health Surveys, and incorporation of datasets from GBD collaborators. The standardized tools used to produce estimates of cirrhosis data by age, sex, location, and year include the cause of death ensemble model (CODEm), spatiotemporal Gaussian process regression (ST-GPR), and DisMod-MR. These three tools are the primary methods employed for this purpose. Detail information on these general GBD methods can be found in earlier publications [3, 13]. The etiological diagnosis of cirrhosis was based on the ICD-10 codes and has been described in earlier studies [2]. The data for this study were obtained from a publicly available database and did not require ethical review or informed consent.

### Variable definition

DALYs for a disease or health condition are the sum of the years of life lost due to premature mortality (YLLs) and the years lived with a disability (YLDs) due to prevalent cases of the disease or health condition in a population. (DALYs = YLLs + YLDs) [15].

According to the WHO definition [16], we divided children and adolescents into four age groups: younger children (aged < 5 years), older children (aged 5–9 years), younger adolescents (aged 10–14 years), and older adolescents (aged 15–19 years) [17].

The SDI (ranging from 0 to 1) was an indicator created by the Institute for Health Metrics and Evaluation (IHME), which reflected the socio-demographic development in the regions. It was calculated from three parts: lag distributed income per capita (LDI), mean education for those age 15 and older (EDU15 +), and total fertility rate under 25 (TFU25). We categorized all countries into 5 level: low, low-middle, middle, high-middle, and high SDI [13].

### Statistical analysis

This statistical analyses consisted of 4 main steps. In step 1, we examined the global trends and every 10 years trends of cirrhosis incidence, prevalence, mortality, and DALYs rate. We used Joinpoint regression model to calculate the annual percent change (APC) and average annual percent change (AAPC) of cirrhosis [18]. Joinpoint regression includes three steps: (1) selecting the model category; (2) determining the number and location of turning points, and (3) optimizing the model. In this study, we choose the log-linear model to describe the changes of rates. The formula was as follows:

$$E\left[\left.{y}_{i}\right|{x}_{i}\right]={e}^{{\beta }_{0}+{\beta }_{1}{x}_{i}+{\delta }_{1}{\left(xi-{\tau }_{1}\right)}^{+}+\cdots +{\delta }_{k}{\left({x}_{i}-{\tau }_{k}\right)}^{+}}$$

For the formula: ${y}_{i}$ dependent variable, ${x}_{i}$ independent variable, ${\beta }_{0}$ invariant parameter, ${\beta }_{1}$ regression coefficient, ${\delta }_{k}$ regression coefficient of segment k piecewise function, ${\tau }_{k}$ unknown inflection point.

The number and location of joinpoints were determined by grid search method, and the maximum number of joinpoints was 5. Monte Carlo permutation test was used to optimize the model. APC, AAPC and 95% confidence interval (CI) were the main outcome indicators of the Joinpoint model.

The formula of APC and the lower and upper confidence interval were as follows:

$$APC=\left[\frac{{y}_{x+1}-{y}_{x}}{{y}_{x}}\right]\times 100=\left({e}^{\beta 1}-1\right)\times 100$$

$$AP{C}_{Lower\left(\alpha \right)}=\left[{e}^{{\beta }_{1}-s\times {t}_{d}^{-1}(1-\alpha /2)}-1\right]\times 100$$

$$AP{C}_{Upper\left(\alpha \right)}=\left[{e}^{{\beta }_{1}+s\times {t}_{d}^{-1}(1-\alpha /2)}-1\right]\times 100$$

For the formulas: $y$ rate of incidence, DALYs, prevalence, and deaths, $x$ year, ${\beta }_{1}$ regression coefficient, $s$ standard error of ${\beta }_{1}$, d is the degree of freedom, ${t}_{d}^{-1}:$ *q* percentile of *t* distribution with degree of freedom *d.*

The formula of AAPC and the lower and upper confidence interval were as follows:

$$ AAPC = \left( {e^{{\Sigma w_{i} \beta_{i} /\Sigma w_{i} }} - 1} \right) \times 100 $$

$$AAP{C}_{Lower\left(\alpha \right)}=\left\{\mathit{exp}\left[\mathit{ln}\left(\left(\frac{AAPC}{100}\right)+1\right)-{Z}_{1-\frac{\alpha }{2}}\sqrt{\sum {\widetilde{\omega }}_{i}^{2}{\sigma }_{i}^{2}}\right]-1\right\}$$

$$AAP{C}_{Lower\left(\alpha \right)}=\left\{\mathit{exp}\left[\mathit{ln}\left(\left(\frac{AAPC}{100}\right)+1\right)+{Z}_{1-\frac{\alpha }{2}}\sqrt{\sum {\widetilde{\omega }}_{i}^{2}{\sigma }_{i}^{2}}\right]-1\right\}$$

For the formulas: ${\omega }_{i}$ the number of years in each segment function, ${\beta }_{i}$ regression coefficient, ${\sigma }_{i}^{2}$ variance

In step 2, we calculated the percentage of relative changes in incident cases and DALYs from 1990 to 2019. What’s more, we reported the incidence, DALYs, prevalence, and death rate of cirrhosis in 1990 and 2019 in global and different subgroups (sex, age, causes). Data were presented as number/rate and 95% uncertainty interval (UI). In step 3, we described the epidemiological trends of cirrhosis in 21 geographical regions, 5 SDI regions, and 204 countries and territories. In step 4, relationship between SDI and incidence and DALYs rates in different regions were also analyzed.

All analyses were performed with R 4.2.1 software and Joinpoint Regression Program (version 4.9.1.0) [19]. p < 0.05 (two sided) were considered statistically significant.

## Results

### Global trends

**Fig. 1:**
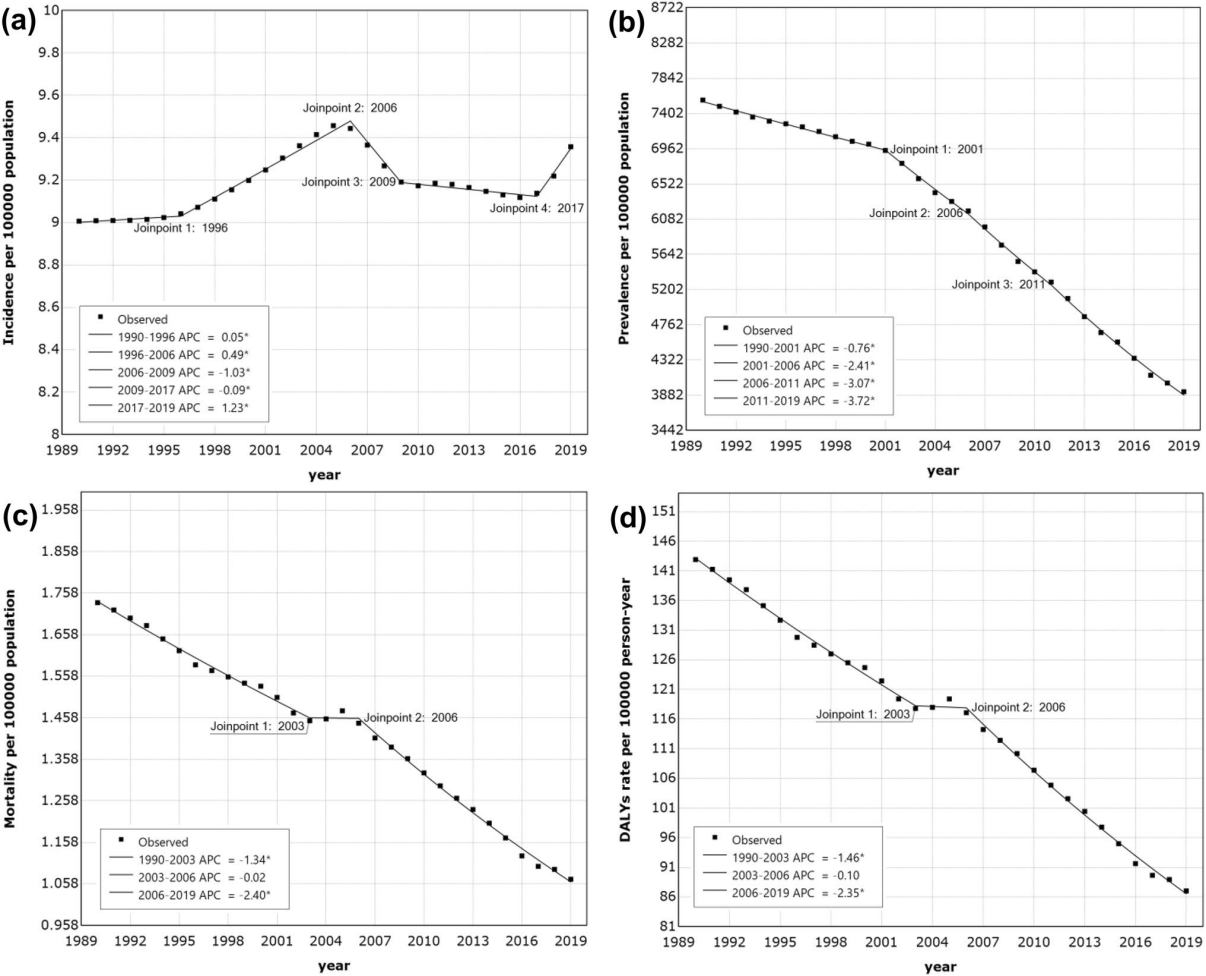
Joinpoint regression analysis of global cirrhosis in children and adolescents from 1990 to 2019. **a** Incidence trends, **b** Prevalence trends, **c** Mortality trends, **d** DALYs trends. *APC* annual percentage change, *DALYs* disability-adjusted life-years

Globally, the cirrhosis incidence increased between 1990 and 1999 (AAPC 0.20 [95% CI 0.17 to 0.23), and continued to increase between 2010 and 2019 (AAPC 0.20 [0.14 to 0.27], Table 1). Overall, incident cases of cirrhosis in children and adolescents increased from 204,767 (141,835 to 275,343) in 1990 to 241,364 (170,007 to 322,891) in 2019, an increase of 17.9% (Table 2). The incidence in 2019 was also higher than 1990 (9.4 [6.6 to 12.5] per 100,000 population vs. 9 [6.2 to 12.1]; AAPC 0.13 [95% CI 0.10 to 0.16]), (Table 2). The joinpoint regression identified a substantial change in incidence of cirrhosis in 1996, 2006, 2009, and 2017 (Fig. 1, Table S1). Prevalence (AAPC = − 2.27 [− 2.39 to − 2.15]), mortality (AAPC = − 1.68 [− 1.86 to − 1.5]), and DALYs rate (AAPC = − 1.72 [− 1.88 to − 1.56]) of cirrhosis have decreased significantly (Table 1). The cases of prevalence, deaths, and DALYs were decreased by 41.2%, 30.1%, and 30.9% between 1990 and 2019, respectively (Table 2, Table S2).

**Table 1:** Global AAPCs in incidence, prevalence, mortality, and DALYs

Time period	Incidence		Prevalence		Mortality		DALYs	
	AAPC (95% CI)	p value	AAPC (95% CI)	p value	AAPC (95% CI)	p value	AAPC (95% CI)	p value
1990–2019	0.13 (0.10 to 0.16)	< 0.001	− 2.27 (− 2.39 to − 2.15)	< 0.001	− 1.68 (− 1.86 to − 1.5)	< 0.001	− 1.72 (− 1.88 to − 1.56)	< 0.001
1990–1999	0.20 (0.17 to 0.23)	< 0.001	− 0.76 (− 0.85 to − 0.66)	< 0.001	− 1.34 (− 1.48 to − 1.21)	< 0.001	− 1.46 (− 1.58 to − 1.33)	< 0.001
2000–2009	− 0.02 (− 0.10 to 0.06)	0.595	− 2.45 (− 2.73 to − 2.18)	< 0.001	− 1.26 (− 1.79 to − 0.72)	< 0.001	− 1.31 (− 1.8 to − 0.81)	< 0.001
2010–2019	0.20 (0.14 to 0.27)	< 0.001	− 3.65 (− 3.80 to − 3.5)	< 0.001	− 2.40 (− 2.49 to − 2.30)	< 0.001	− 2.35 (− 2.43 to − 2.27)	< 0.001

*AAPC* average annual percentage changes, *DALYs* disability-adjusted life-years

**Table 2:** Incidence and DALYs of cirrhosis in children and adolescents at global and regional levels

Subgroup	Incidence					DALYs				
	No., 1990	No. 2019	Rate 1990 (/10^5)	Rate 2019 (/10^5)	No. change (%)	No. 1990	No. 2019	Rate 1990 (/10^5)	Rate 2019 (/10^5)	No. change (%)
Global	204,767 (141,835–275,343)	241,364 (170,007–322,891)	9 (6.2–12.1)	9.4 (6.6–12.5)	17.9	3,249,018 (2,365,190–4,332,170)	2,244,642 (1,849,618–2,724,604)	142.9 (104–190.5)	87 (71.7–105.6)	− 30.9
Sex										
Female	98,110 (68,609–131,228)	117,053 (82,656–155,042)	8.8 (6.2–11.8)	9.4 (6.6–12.4)	19.3	1,595,229 (1,108,257–2,193,196)	1,004,207 (813,079–1,225,332)	143.9 (100–197.8)	80.3 (65.1–98)	− 37.0
Male	106,657 (73,338–144,874)	124,311 (86,214–168,746)	9.2 (6.3–12.4)	9.4 (6.5–12.7)	16.6	1,653,789 (1,212,232–2,249,569)	1,240,434 (1,003,225–1,527,754)	142 (104.1–193.1)	93.3 (75.5–114.9)	− 25.0
Age										
< 5 years	96,858 (57,908–139,333)	95,611 (55,699–140,591)	15.3 (9.2–22)	14.4 (8.4–21.2)	− 1.3	1,389,082 (801,290–2,107,269)	649,882 (475,582–894,599)	219.8 (126.8–333.4)	98 (71.7–135)	− 53.2
5–9 years	42,367 (20,143–70,514)	51,712 (27,157–83,422)	7.2 (3.4–12.1)	7.9 (4.1–12.7)	22.1	626,707 (448,627–830,306)	396,933 (309,548–498,139)	107.1 (76.7–141.9)	60.6 (47.3–76.1)	− 36.7
10–14 years	30,240 (11,435–53,666)	43,718 (19,054–74,148)	5.6 (2.1–10)	6.8 (3–11.5)	44.6	446,404 (360,635–534,044)	394,251 (327,878–470,997)	83.2 (67.2–99.5)	61.4 (51.1–73.3)	− 11.7
15–19 years	35,301 (11,458–67,013)	50,323 (18,776–90,640)	6.8 (2.2–12.9)	8.1 (3–14.6)	42.6	786,826 (681,204–911,492)	803,575 (690,625–932,179)	151.4 (131.1–175.4)	129.7 (111.5–150.5)	2.1
Cause										
Alcohol use	127 (22–362)	188 (34–525)	0 (0–0)	0 (0–0)	48.0	2901 (922–6465)	2904 (910–6301)	0.1 (0–0.3)	0.1 (0–0.2)	0.1
Hepatitis B	4751 (2186–9173)	4879 (2198–9464)	0.2 (0.1–0.4)	0.2 (0.1–0.4)	2.7	91,675 (51,406–142,993)	67,798 (38,552–105,829)	4 (2.3–6.3)	2.6 (1.5–4.1)	− 26.0
Hepatitis C	4773 (2324–8890)	6143 (2951–11,436)	0.2 (0.1–0.4)	0.2 (0.1–0.4)	28.7	89,441 (50,416–146,065)	79,335 (46,094–125,660)	3.9 (2.2–6.4)	3.1 (1.8–4.9)	− 11.3
NAFLD	833 (202–2079)	1081 (297–2684)	0 (0–0.1)	0 (0–0.1)	29.8	19,138 (7818–37,586)	19,170 (8002–37,918)	0.8 (0.3–1.7)	0.7 (0.3–1.5)	0.2
Other causes	194,283 (135,714–259,675)	229,073 (161,831–304,209)	8.5 (6–11.4)	8.9 (6.3–11.8)	17.9	3,045,863 (2,198,226–4,083,545)	2,075,434 (1,685,561–2,544,092)	134 (96.7–179.6)	80.5 (65.4–98.6)	− 31.9
SDI										
Low SDI	37,711 (27,147–49,708)	76,008 (54,591–99,841)	12.8 (9.2–16.8)	12.7 (9.1–16.7)	101.6	726,230 (452,802–1,084,596)	877,010 (682,513–1,118,226)	245.9 (153.3–367.2)	146.9 (114.3–187.3)	20.8
Low-middle SDI	69,224 (49,774–90,930)	83,806 (59,283–112,589)	12.1 (8.7–15.9)	12.1 (8.5–16.2)	21.1	1,298,381 (907,080–1,751,523)	810,843 (651,663–999,528)	227.6 (159–307)	116.6 (93.7–143.7)	− 37.5
Middle SDI	62,016 (42,241–84,777)	53,834 (37,271–72,567)	8.1 (5.5–11.1)	7.3 (5.1–9.9)	− 13.2	940,701 (722,297–1,204,038)	450,209 (378,530–539,047)	122.7 (94.2–157)	61.1 (51.4–73.2)	− 52.1
High-middle SDI	25,283 (16,039–35,850)	19,741 (12,808–27,756)	6.2 (4–8.8)	6 (3.9–8.5)	− 21.9	235,034 (197,189–280,658)	89,491 (77,510–103,596)	58 (48.7–69.3)	27.3 (23.6–31.6)	− 61.9
High SDI	10,452 (6434–15,041)	7881 (4978–11,126)	4.5 (2.7–6.4)	3.6 (2.3–5)	− 24.6	46,672 (42,894–50,891)	15,801 (13,489–18,445)	19.9 (18.3–21.7)	7.2 (6.1–8.4)	− 66.1
Region										
Andean Latin America	1669 (1252–2096)	1442 (1032–1904)	8.7 (6.5–11)	6.1 (4.4–8)	− 13.6	46,879 (33,852–68,498)	12,309 (8967–16,550)	245 (176.9–358)	52 (37.9–69.9)	− 73.7
Australasia	101 (47–162)	106 (55–162)	1.6 (0.8–2.6)	1.5 (0.8–2.2)	5.0	371 (313–436)	218 (172–276)	5.9 (5–6.9)	3 (2.4–3.8)	− 41.2
Caribbean	883 (613–1160)	796 (522–1108)	5.9 (4.1–7.7)	5.1 (3.4–7.1)	− 9.9	25,541 (14,627–41,699)	13,077 (7012–21,328)	169.3 (96.9–276.4)	84.2 (45.1–137.3)	− 48.8
Central Asia	3753 (2878–4766)	4497 (3307–5948)	11.9 (9.1–15.1)	13.2 (9.7–17.5)	19.8	50,193 (44,785–56,607)	36,974 (32,163–42,534)	159.1 (142–179.5)	108.8 (94.6–125.2)	− 26.3
Central Europe	2316 (1424–3283)	1526 (968–2163)	6 (3.7–8.5)	6.5 (4.1–9.2)	− 34.1	17,580 (15,723–20,576)	3737 (3156–4374)	45.6 (40.8–53.4)	16 (13.5–18.7)	− 78.7
Central Latin America	5034 (3251–6873)	3406 (2067–4773)	6.1 (4–8.4)	3.9 (2.4–5.5)	− 32.3	98,567 (88,188–110,979)	39,179 (32,423–47,173)	119.8 (107.2–134.9)	44.8 (37.1–53.9)	− 60.3
Central Sub-Saharan Africa	4265 (3219–5467)	10,012 (7492–13,039)	13.5 (10.2–17.3)	14.1 (10.5–18.3)	134.7	69,614 (46,971–102,194)	90,003 (49,975–161,426)	220.9 (149–324.2)	126.5 (70.2–226.8)	29.3
East Asia	22,580 (13,181–33,785)	8181 (4591–12,312)	4.9 (2.8–7.3)	2.6 (1.5–4)	− 63.8	204,684 (159,947–248,524)	30,050 (25,158–35,716)	44 (34.4–53.4)	9.7 (8.1–11.5)	− 85.3
Eastern Europe	4795 (3074–6903)	4686 (2940–6774)	7.1 (4.6–10.3)	9.9 (6.2–14.3)	− 2.3	20,694 (18,892–23,377)	9890 (8639–11,372)	30.7 (28.1–34.7)	20.9 (18.2–24)	− 52.2
Eastern Sub-Saharan Africa	10,945 (7719–14,657)	22,174 (15,512–29,881)	9.9 (7–13.2)	9.9 (6.9–13.4)	102.6	221,678 (132,258–320,003)	253,138 (173,757–353,166)	200.4 (119.5–289.3)	113.2 (77.7–158)	14.2
High-income Asia Pacific	3952 (2564–5551)	2212 (1458–3033)	7.8 (5.1–11)	6.8 (4.5–9.4)	− 44.0	16,981 (14,710–18,816)	2743 (2162–3499)	33.7 (29.2–37.3)	8.5 (6.7–10.8)	− 83.8
High-income North America	1924 (1041–3075)	1293 (768–1986)	2.4 (1.3–3.8)	1.4 (0.9–2.2)	− 32.8	11,109 (10,081–11,990)	5242 (4676–5660)	13.6 (12.4–14.7)	5.8 (5.2–6.3)	− 52.8
Mexico	3108 (1971–4395)	1868 (1174–2601)	7.2 (4.5–10.1)	4.3 (2.7–6)	− 39.9	43,334 (35,643–51,417)	17,633 (14,955–20,405)	99.8 (82–118.4)	40.8 (34.6–47.2)	− 59.3
North Africa and Middle East	16,869 (12,454–22,015)	21,527 (15,623–28,267)	9.4 (6.9–12.2)	9.4 (6.8–12.4)	27.6	309,785 (179,653–458,202)	186,856 (147,215–238,429)	171.8 (99.6–254.1)	81.7 (64.3–104.2)	− 39.7
Oceania	126 (78–181)	214 (124–313)	3.8 (2.4–5.5)	3.5 (2–5.1)	69.8	2434 (1762–3189)	3503 (2473–4946)	73.5 (53.2–96.3)	57.4 (40.5–81)	43.9
South Asia	82,053 (58,014–108,589)	103,650 (73,158–139,870)	15 (10.6–19.8)	14.9 (10.5–20.1)	26.3	1,290,375 (874,232–1,756,539)	846,866 (669,614–1,047,589)	235.4 (159.5–320.5)	121.9 (96.4–150.8)	− 34.4
Southeast Asia	16,353 (10,229–23,747)	10,441 (6523–15,193)	7.4 (4.6–10.7)	4.6 (2.9–6.7)	− 36.2	466,983 (284,869–687,208)	210,714 (179,092–248,201)	211 (128.7–310.6)	93.5 (79.4–110.1)	− 54.9
Southern Latin America	621 (338–948)	723 (407–1080)	3.2 (1.7–4.9)	3.6 (2–5.4)	16.4	7572 (6775–8380)	3632 (3050–4253)	39.1 (35–43.3)	18.2 (15.3–21.3)	− -52.0
Southern Sub-Saharan Africa	2846 (1975–3858)	2600 (1763–3569)	10.9 (7.5–14.7)	8.5 (5.8–11.7)	− 8.6	19,553 (14,704–25,386)	11,995 (8711–16,092)	74.6 (56.1–96.9)	39.2 (28.5–52.6)	− 38.7
Tropical Latin America	3321 (1981–4878)	1659 (970–2473)	4.8 (2.8–7)	2.5 (1.5–3.7)	− 50.0	61,748 (54,074–71,622)	17,964 (15,824–20,322)	88.7 (77.7–102.9)	26.9 (23.7–30.4)	− 70.9
Western Europe	4355 (2453–6630)	3707 (2136–5579)	4.4 (2.5–6.7)	4 (2.3–6)	− 14.9	14,628 (13,060–16,619)	5382 (4400–6694)	14.9 (13.3–16.9)	5.8 (4.8–7.3)	− 63.2
Western Sub-Saharan Africa	16,007 (11,695–20,953)	36,514 (26,107–47,836)	14.9 (10.9–19.5)	14.7 (10.5–19.3)	128.1	292,047 (202,711–429,062)	461,169 (337,054–611,591)	272.1 (188.8–399.7)	185.8 (135.8–246.3)	57.9

*DALYs* disability-adjusted life-years, *SDI* socio-demographic index

### Global trends by sex

**Fig. 2:**
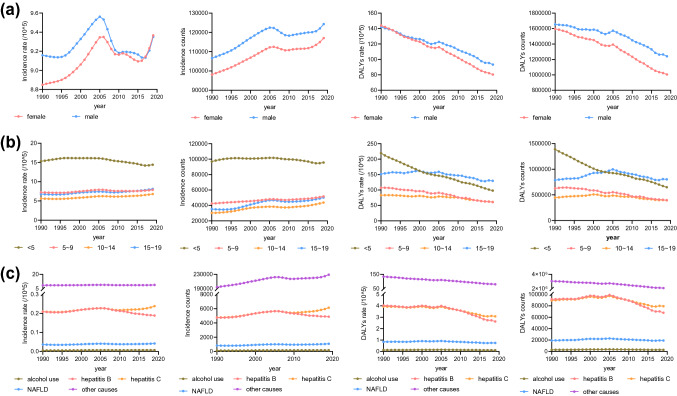
Incidence and DALYs of cirrhosis in subgroups in children and adolescents from 1990 to 2019. **a** Sex subgroup, **b** Age subgroup, **c** Causes subgroup

Although both male (AAPC = 0.1, p < 0.001) and female (AAPC = 0.2, p < 0.001) incidence rates were increasing from1990 to 2019, female was slightly higher than male (Table 3). From 1990 to 2019, the incident number of male and female increased by 16.9% and 19.3%, respectively (Table 2). The changes of incidence rate of male and female were similar, with a trend of “rising–falling–rising again” (Fig. 2). Around 2005, the incidence rate of both male and female reached the peak, and since 2017, the incidence rate has gradually increased again. The number of patients, both male and female, increased year by year (Fig. 2).

**Table 3:** Subgroups AAPCs in incidence and DALYs

	Incidence		DALYs	
	AAPC (95% CI)	p value	AAPC (95% CI)	p value
Sex				
Female	0.2 (0.2 to 0.2)	< 0.001	− 2 (− 2.2 to − 1.8)	< 0.001
Male	0.1 (0 to 0.1)	< 0.001	− 1.4 (− 1.6 to − 1.2)	< 0.001
Age				
< 5 years	− 0.2 (− 0.3 to − 0.2)	< 0.001	− 2.8 (− 2.9 to − 2.7)	< 0.001
5–9 years	0.3 (0.3 to 0.3)	< 0.001	− 2.1 (− 2.2 to − 1.9)	< 0.001
10–14 years	0.6 (0.6 to 0.7)	< 0.001	− 1.1 (− 1.2 to − 1)	< 0.001
15–19 years	0.6 (0.6 to 0.7)	< 0.001	− 0.7 (− 0.8 to − 0.6)	< 0.001
Cause				
Alcohol use	1 (0.8 to 1.1)	< 0.001	− 0.4 (− 0.8 to 0)	0.047
Hepatitis B	− 0.3 (− 0.4 to − 0.2)	< 0.001	− 1.5 (− 1.7 to − 1.2)	< 0.001
Hepatitis C	0.4 (0.4 to 0.5)	< 0.001	− 0.8 (− 1 to − 0.6)	< 0.001
NAFLD	0.5 (0.3 to 0.6)	< 0.001	− 0.4 (− 0.7 to − 0.2)	0.002
Other causes	0.1 (0.1 to 0.2)	< 0.001	− 1.8 (− 1.9 to − 1.6)	< 0.001
SDI				
Low SDI	0 (0 to 0)	0.146	− 1.7 (− 1.9 to − 1.6)	< 0.001
Low-middle SDI	0 (− 0.1 to 0)	0.008	− 2.3 (− 2.5 to − 2.1)	< 0.001
Middle SDI	− 0.4 (− 0.4 to − 0.3)	< 0.001	− 2.4 (− 2.7 to − 2.1)	< 0.001
High-middle SDI	− 0.1 (− 0.2 to 0)	0.005	− 2.6 (− 2.9 to − 2.3)	< 0.001
High SDI	− 0.8 (− 0.8 to − 0.7)	< 0.001	− 3.5 (− 3.7 to − 3.2)	< 0.001
Region				
Andean Latin America	− 1.3 (− 1.3 to − 1.2)	< 0.001	− 5.2 (− 5.5 to − 4.9)	< 0.001
Australasia	− 0.3 (− 0.3 to − 0.3)	< 0.001	− 2.3 (− 2.9 to − 1.8)	< 0.001
Caribbean	− 0.5 (− 0.5 to − 0.4)	< 0.001	− 2.4 (− 2.6 to − 2.2)	< 0.001
Central Asia	0.3 (0.2 to 0.5)	< 0.001	− 1.3 (− 1.7 to − 0.9)	< 0.001
Central Europe	0.3 (0.3 to 0.4)	< 0.001	− 3.6 (− 4 to − 3.2)	< 0.001
Central Latin America	− 1.5 (− 1.6 to − 1.5)	< 0.001	− 3.3 (− 3.5 to − 3)	< 0.001
Central Sub-Saharan Africa	0.1 (0.1 to 0.2)	< 0.001	− 1.9 (− 2.1 to − 1.7)	< 0.001
East Asia	− 2.1 (− 2.3 to − 1.8)	< 0.001	− 5.2 (− 5.6 to − 4.8)	< 0.001
Eastern Europe	1.2 (1.1 to 1.3)	< 0.001	− 1.4 (− 2.3 to − 0.5)	0.003
Eastern Sub-Saharan Africa	0 (0 to 0)	0.252	− 2 (− 2.1 to − 1.9)	< 0.001
High-income Asia Pacific	− 0.5 (− 0.5 to − 0.4)	< 0.001	− 4.6 (− 4.9 to − 4.4)	< 0.001
High-income North America	− 1.7 (− 1.9 to − 1.5)	< 0.001	− 2.9 (− 3.3 to − 2.5)	< 0.001
Mexico	− 1.7 (− 1.9 to − 1.6)	< 0.001	− 2.9 (− 3.2 to − 2.6)	< 0.001
North Africa and Middle East	0 (0 to 0)	0.27	− 2.5 (− 2.7 to − 2.3)	< 0.001
Oceania	− 0.3 (− 0.3 to − 0.3)	< 0.001	− 0.9 (− 1.2 to − 0.5)	< 0.001
South Asia	0 (− 0.1 to 0)	0.706	− 2.2 (− 2.7 to − 1.8)	< 0.001
Southeast Asia	− 1.6 (− 1.8 to − 1.4)	< 0.001	− 2.8 (− 2.9 to − 2.6)	< 0.001
Southern Latin America	0.4 (0.3 to 0.5)	< 0.001	− 2.6 (− 3.1 to − 2.1)	< 0.001
Southern Sub-Saharan Africa	− 0.8 (− 0.9 to − 0.8)	< 0.001	− 2.2 (− 2.8 to − 1.5)	< 0.001
Tropical Latin America	− 2.2 (− 2.4 to − 1.9)	< 0.001	− 4.1 (− 4.3 to − 3.8)	< 0.001
Western Europe	− 0.3 (− 0.4 to − 0.3)	< 0.001	− 3.2 (− 3.6 to − 2.8)	< 0.001
Western Sub-Saharan Africa	0 (− 0.1 to 0)	0.012	− 1.3 (− 1.5 to − 1.1)	< 0.001

*DALYs* disability-adjusted life-years, *AAPC* average annual percentage change, *SDI* socio-demographic index

Both DALYs count and DALYs rate decreased significantly (Fig. 2), but the decline in female was higher than male (AAPC − 2 [95% CI − 2.2 to − 1.8] vs. − 1.4 95% CI [− 1.6 to − 1.2], Table 3). The prevalence rate (AAPC − 2.3 and − 2.2, all p < 0.001) and mortality (AAPC − 2 and − 1.4, all p < 0.001) of cirrhosis decreased significantly in both female and male, and more significantly in female than in male (Table S3, Figure S1).

### Global trends by age group

The incident cases in younger children (< 5 years old) were significantly higher than any other age groups, reaching 95,611 (55,699 to 140,591) in 2019 (Table 2). The incidence rate of younger children was also highest (14.4 [8.4–21.2] per 100,000 population). However, from the trend of incidence rate, except for younger children (AAPC = − 0.2, p < 0.001), other age groups have significantly increased (Table 3). AAPC was 0.3, 0.6, and 0.6 in older children, younger adolescents, and older adolescents (all p < 0.001, Table 3). The number of cirrhosis in older children (42,367 in 1990 to 51,712 in 2019), younger adolescents (30,240 in 1990 to 43,718 in 2019) and older adolescents (35,301 in 1990 to 50,323 in 2019) increased by 22.1%, 44.6% and 42.6% respectively, while young children (96,858 in 1990 to 95,611 in 2019) decreased by 3% (Table 2, Fig. 2).

DALY counts decreased in all age groups, among which young children (1,389,082 in 1990 to 649,882 in 2019) dropped most significantly by 53.2%, except for older adolescents (increased 2.1%, Table 2). Prevalence and mortality rates have also decreased significantly (Table S2, Table S3, Figure S1).

### Global trends by causes

From 1990 to 2019, the number of cirrhosis caused by 5 etiologies (alcohol use, hepatitis B, hepatitis C, NAFLD, and other causes) increased, among which alcohol use was most obvious, with an increase of 48%, followed by hepatitis C and NAFLD, with 29.8% and 28.7%, respectively (Table 2). It was worth noting that since about 2010, the cirrhosis caused by hepatitis B has shown downward trends, while hepatitis C has shown upward trends (Fig. 2). Joinpoint regression indicated that incidence rate of alcohol use (AAPC = 1, p < 0.001), NAFLD (AAPC = 0.5, p < 0.001) and hepatitis C (AAPC = 0.4, p < 0.001) were increasing, while only hepatitis B (AAPC = − 0.3, p < 0.001) decreasing (Table 3).

The DALY count of alcohol use and NAFLD increased by 0.1% and 0.2%; while DALY count of hepatitis B and hepatitis C decreased by 26% and 11.3% (Table 2). Joinpoint regression showed that the DALY rate of all causes decreased over the 30 years, the most obvious being hepatitis B (AAPC = − 1.5, p < 0.001, Table 3) The prevalence and mortality of both hepatitis B and hepatitis C were reduced, in the past three decades (Table S2, Table S3, Figure S1).

### Global trends by SDI

The incidence cases of cirrhosis were increased in low (101.6%) and low-middle SDI (21.1%) areas, while decreasing in middle and above SDI areas (Table 2). In 2019, the incidence cases were about 10 times in high SDI (7881 [95% CI 4978 to 11126]) areas compared with low (76,008 [95% CI 54,591 to 99841]) or low-middle SDI (83,806 [95% CI 59,283 to 112589]) areas. The AAPC of incidence rate in low-middle and above SDI areas were decreasing, while there was no significant change in low SDI areas (AAPC = 0, p = 0.146, Table 3). The DALY count also increased only in low SDI areas (20.8%), while decreased in other areas (Table 2). In 2019, the DALY rate of low and low-middle SDI areas (146.9 and 116.6 per 100,000 person-year) were more than 15 times higher than high SDI areas (7.2 per 100,000 person-year). The joinpoint regression indicated that the DALY rates in all regions have been decreasing (all AAPC < 0, all p < 0.001, Table 3). Prevalence and deaths were shown in Table S2 and Table S3.

## Regional trends

At the regional level, the largest increases count in cirrhosis between 1990 and 2019 was observed in Central Sub-Saharan Africa (increased by 134.7%, from 4265 in 1990 to 10,012 in 2019), Western Sub-Saharan Africa (increased by 128.1%, from 16,007 in 1990 to 36,514 in 2019), and Eastern Sub-Saharan Africa (increased by 102.6%, from 10,945 in 1990 to 22,174 in 2019, Table 2). The highest incidence rate in 2019 was in South Asia (14.9 per 100,000 population) and Western Sub-Saharan Africa (14.7 per 100,000 population). The number of cases in East Asia and Tropical Latin America decreased by more than 50%. The joinpoint regression implied that only 5 of the 21 regions had a significant increase in incidence rate, which were Eastern Europe (AAPC = 1.2), Southern Latin America (AAPC = 0.4), Central Asia (AAPC = 0.3), Central Europe (AAPC = 0.3), Central Sub-Saharan Africa (AAPC = 0.1) in turn (all p < 0.001), while the other regions had a declined trends or unchanged (Table 3).

Among the 21 regions, DALY counts were increasing only in 4 regions (Western Sub-Saharan Africa, Oceania, Central Sub-Saharan Africa, and Eastern Sub-Saharan Africa), of which West Africa (57.9%) has the largest increase (Table 2). In the past three decades, the DALYs rate has decreased year by year in all regions (all AAPC < 0, all p < 0.001, Table 3), the most significant being Andean Latin America (AAPC = − 5.2) and East Asia (AAPC = − 5.2). The regional prevalence and mortality were shown in Table S2 and Table S3.

### National trends

**Fig. 3:**
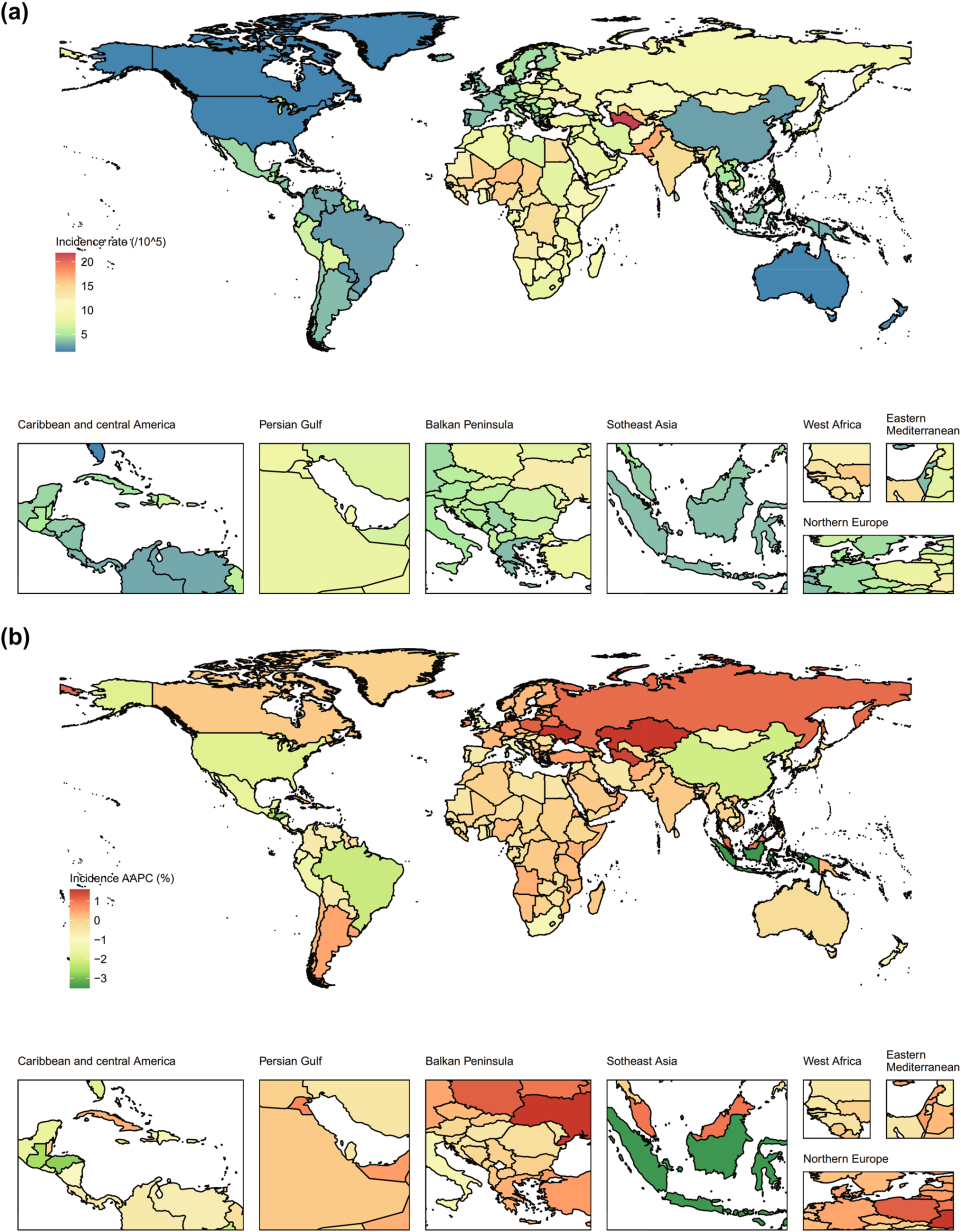
Geographical distribution of cirrhosis incidence in children and adolescents in 204 countries and territories. **a** Incidence of cirrhosis in 2019, **b** AAPC in incidence of cirrhosis between 1990 and 2019. *AAPC* average annual percentage change

At the national level, the highest incidence rate and most cases of cirrhosis were in Turkmenistan (21.8 [95% CI 16.0 to 28.5] per 100,000 population) and India (75,299 [95% CI 102,573 to 52,314), respectively in 2019 (Fig. 3). In the past 30 years, the most pronounced increase in the incidence rate of cirrhosis between 1990 and 2019 were in Kazakhstan (AAPC = 1.6, p < 0.001), Ukraine (AAPC = 1.6, p < 0.001), and Turkmenistan (AAPC = 1.5, p < 0.001, Fig. 3). From 1990 to 2019, the number of incident cases increased by more than 200% in 3 countries, Afghanistan (276%), Angola (234%), and Somalia (201%, Figure S2).

**Fig. 4:**
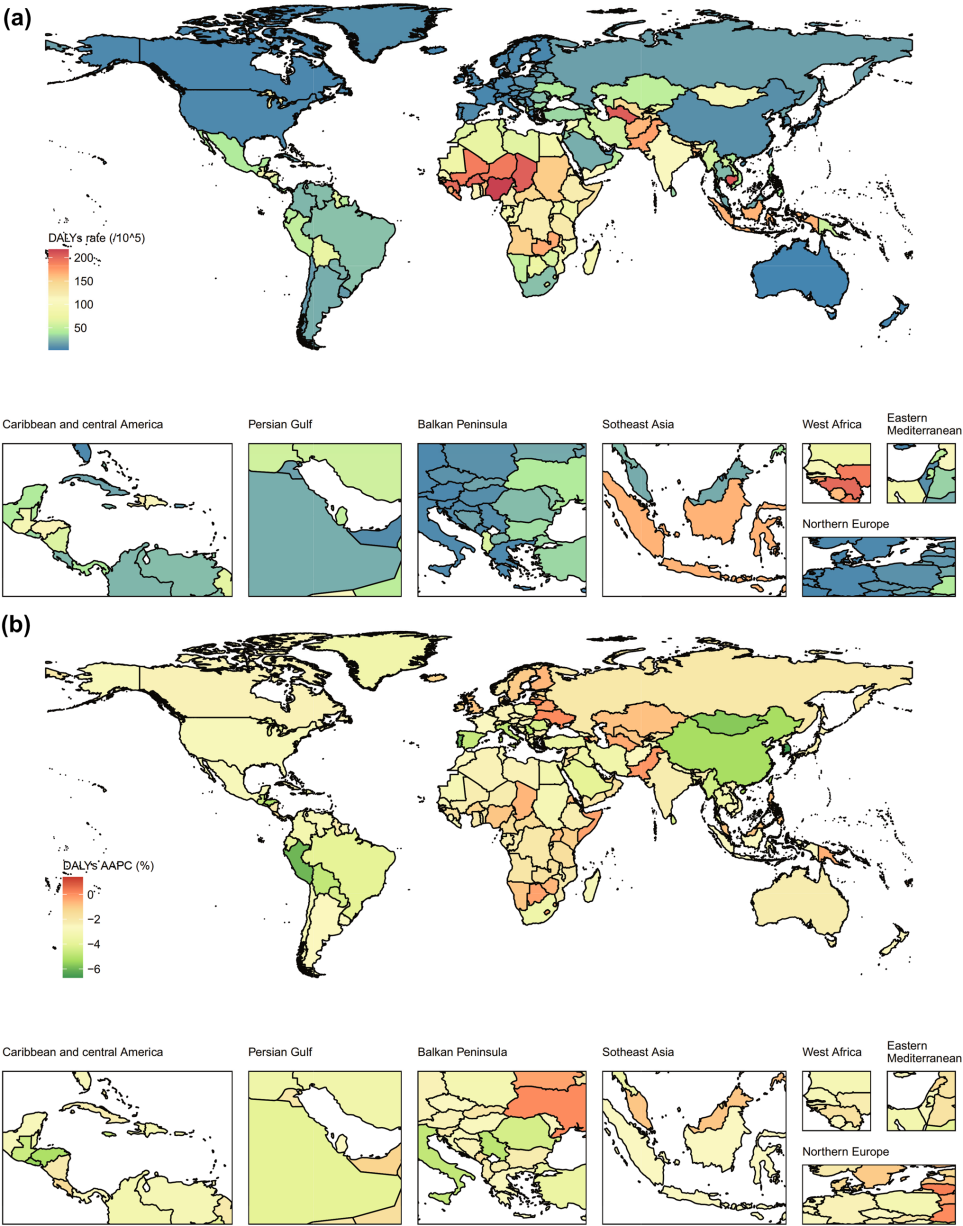
Geographical distribution of cirrhosis DALYs in children and adolescents in 204 countries and territories. **a** DALYs rate of cirrhosis in 2019, **b** AAPC in DALYs rate of cirrhosis between 1990 and 2019.DALYs: disability-adjusted life-years, *AAPC* average annual percentage change

In 2019, Nigeria (217.6 [95% CI 140.6 to 328.6]) and India (539,837 [95% CI 417,682 to 696702]) have the highest DALYs rate and DALYs count (Fig. 4). Among 204 countries and territories, only Armenia DALY rate (AAPC = 1.4, p < 0.001) has been rising since 1990 to 2019, while the rest declined or without significant changes (Fig. 4). Between 1990 and 2019, the largest increase rate in DALYs count was observed in Somalia (155%) and Chad (132%, Figure S2). National prevalence and mortality of cirrhosis, and the associated AAPCs between 1990 and 2019, are in Figure S2, Figure S3, and Figure S4.

### Trends between SDI and cirrhosis

**Fig. 5:**
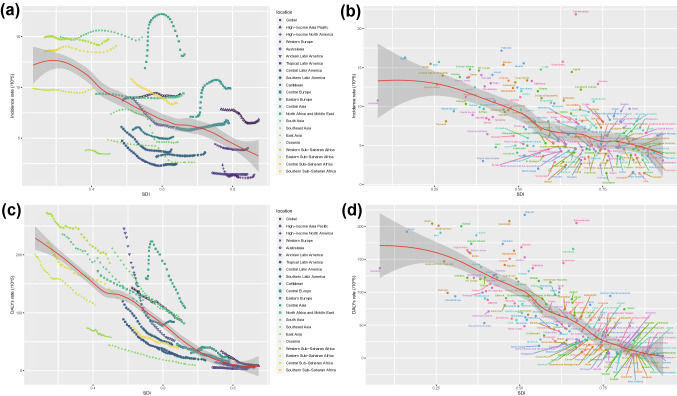
Global, 21 regions, and 204 countries and territories burden of cirrhosis in children and adolescents by SDI, from 1990 to 2019. **a** Incidence per 100,000 population in global and 21 regions, **b** Incidence per 100,000 population in global and 204 countries and territories, **c** DALYs per 100,000 population in global and 21 regions, **d** DALYs per 100,000 population in global and 204 countries and territories. For each region, points from left to right depict estimates from each year from 1990 to 2019. Expected trends based on SDI and disease rates in all locations were shown as the red line with LOWESS (locally weighted scatterplot smoothing) methods. *DALYs* disability-adjusted life-years, *SDI* socio-demographic index

Incidence of cirrhosis was significant higher at lower SDI levels, especially in Sub-Saharan Africa (Fig. 5). However, Central Asia was opposite, with medium SDI but high incidence rate. The incidence rate has a plateau near SDI 0.7, because the incidence rate of Eastern Europe has suddenly increased. In global, DALYs rate and SDI showed an obvious negative correlation (Fig. 5). The DALYs rate in almost regions has declined year by year, except Central Asia, which experienced a change of rising and then declining. Figure 5 right panel showed the relationship between incidence rate and SDI, and the relationship between DALYs rate and SDI in 204 countries and territories.

## Discussion

To our knowledge, this was the first study to describe incidence, prevalence, deaths, and DALYs changes of cirrhosis among children and adolescents aged less than 19 years, from 1990 to 2019, at the global, regional, and national levels. Globally, the incidence rates of cirrhosis have been increasing, while the DALYs rates have been decreasing since 1990 to 2019. The annual incidence rate of female was higher than male. Except for younger children, the incidence rates of other ages have increased significant (AAPC > 0). In terms of etiology, except for hepatitis B, cirrhosis of hepatitis C, alcohol use and NAFLD have increased significantly. Except for low SDI areas, the incidence rate has declined year by year in other areas. We noted that SDI was negatively correlated with incidence rate. At the regional level, the incidence rates varied greatly among regions. It was gratifying to note that the DALYs rates of all sexes, ages, causes and regions have been decreasing year by year (all AAPC < 0), although the degree of decline was different.

Although the incidence rate of both male and female in 2019 was 9.4 per 100,000 population, the increasing rate of female was higher than male from 1990 to 2019, among children and adolescents. However, the global cirrhosis burden of adult (20 + years) in male was 1.5 times higher than female [14]. In a retrospective cohort, which included 16,738 patients with cirrhosis caused by hepatitis B from 2008 to 2015 in China, male patients were 1.8 times than female [20]. The gender difference in the incidence rate of cirrhosis in children and adolescents seemed not obvious than adults. DALYs rate of male was higher than female, which was consistent with GBD 2017 Cirrhosis Collaborators study [2].

It was worth noting that only the incidence rate of cirrhosis in children under 5 years old decreased year by year, while the other age groups increased with each passing year. This may be related to the decline in the prevalence of hepatitis B. It is estimated that there was a marked decline of 76·8% (76.2 to 77.5) in prevalence in children less than 5 years between 1990 and 2019 [3]. The increase in incidence rate in other age groups may be attributed to hepatitis C, alcohol use and NAFLD.

Among all the causes of cirrhosis, only the incidence rate of hepatitis B was decreasing (AAPC < 0). However, it was still one of the main etiologies of cirrhosis. The significant decline in the incidence of hepatitis B was mainly attributable to the widespread availability of the hepatitis B vaccine. Taking China as an example, since hepatitis B vaccine was incorporated into children's immunization plan in 1992, the positive rate of HBsAg among children under 5 years old has dropped from 9.9% in 1992 to 0.3% in 2014. In 2015, the coverage rate of three doses of children's vaccine in China has reached 99.58% [21]. In May 2016, WHO proposed a goal that elimination of viral hepatitis as a public health threat by 2030 (defined as a 65% reduction in mortality and a 90% reduction in morbidity compared with 2015) [22]. Timely antiviral treatment can significantly reduce hepatitis B related deaths [23]. Although achieving clinical cure (HBsAg loss, with or without anti-HBs seroconversion) of hepatitis B was not easy at least for the moment, oral antiviral treatment (fist line ETV, TDF, and TAF) can still effectively reduce the incidence of liver cancer in patients [24]. Unfortunately, WHO estimated that only 17% patients diagnosed with hepatitis B have received treatment, and only 10% of patients know that they were infected with HBV [22].

The global estimate for viremic prevalence in the pediatric population aged 0–18 years was 0.13%, corresponding to 3.26 million children with HCV in 2018 [12]. The prevention of hepatitis C was similar with hepatitis B, but the injecting drugs among adolescents needed special attentions. The proportion of HCV infections in people who inject drugs was significantly associated with HCV prevalence in children aged 15–19 years [12]. Although there were available oral drugs (direct antiviral agent, DAA) to achieve clinical cure for hepatitis C, the awareness rate (19%) and treatment rate (only 5 million) of hepatitis C were still low [22].

Our results showed that the incidence and prevalence of NAFLD in children and adolescents was increasing (all AAPC > 0), which were consistent with the growth trend of adults. One quarter of the global population was estimated to have NAFLD [25]. A meta-analysis in children and adolescents (74 studies, 276,091 participants) showed that overall prevalence of NAFLD was 7.40% (95% CI 4.17–12.81) regardless of the diagnostic method [26]. Different from adults, our research showed that the mortality or DALYs of cirrhosis caused by NAFLD was decreasing (all AAPC < 0), while adults were increasing year by year [25]. HCC caused by NAFLD mainly in the elderly (mean age 73 years) may explain the difference between them [27].

The annual incidence growth rate of cirrhosis caused by alcohol abuse was the highest among all causes in our study (AAPC maximum). Worldwide, more than a quarter of all people aged 15–19 years were current drinkers, amounting to 155 million adolescents. Prevalence of heavy episodic drinking among adolescents aged 15–19 years was 13.6% in 2016, with males most at risk [28]. For adolescents and children, it was crucial to reduce the accessibility of alcohol. WHO has proposed an action “Towards an action plan (2022–2030) to effectively implement the Global strategy to reduce the harmful use of alcohol.” in 2022 [29].

We found regional and national disparity in the incidence rate of cirrhosis in children and adolescents. In low SDI regions or countries, the morbidity and mortality of cirrhosis were higher than other regions or countries. Only low SDI regions did not show a decrease year by year. It was worth noting that the cirrhosis in East Asia was significantly reduced. This may be due to the widespread vaccination of hepatitis B vaccine in China and other countries.

Our study had several limitations. First, although GBD 2019 has used all available data as far as possible, the data was non-available in many countries in low-income and middle-income countries yet. Second, this study did not distinguish whether the cirrhosis was in the compensated or decompensated phase, which has considerable implications for the assessment of mortality risk and DALYs. Third, rates of cirrhosis caused by alcohol abuse and NAFLD were probably underestimated worldwide, because GBD did not provide data for age under 14. Fourth, inherited metabolic liver diseases were the important cause of pediatric cirrhosis. However, because the GBD 2019 did not list these types of causes separately, but instead classifies them under “other causes”. It was impossible to estimate the trend of liver cirrhosis caused by these categories. Nevertheless, this did not affect the analysis of the overall trend of pediatric liver cirrhosis. Fifth, despite multiple appropriate statistical methods were used to estimate the mortality and incidence rates of cirrhosis, the incomplete death registrations and limitations in data quality in developing regions may lead to underestimation of the mortality and incidence rates in these areas.

## Conclusions

In conclusion, the global incidence rate of cirrhosis has been increasing from 1990 to 2019, while the DALYs rate has been decreasing in children and adolescents. Only the incidence rate of children under 5 years old decreased, which may be related to the widespread vaccination of hepatitis B vaccine, while other age groups (5–19 years) increased. Incidence rate of cirrhosis caused by hepatitis B declined, while the proportion of hepatitis C, NAFLD, and alcohol use increased. We appealed for that in low SDI regions and countries, promoting the widespread vaccination of hepatitis B vaccine and reducing HCV infection were prominent; in middle and high SDI regions and countries, reducing cirrhosis caused by NAFLD and alcohol use were urgent.

### Supplementary Information

Below is the link to the electronic supplementary material.

Supplementary file1 (PDF 10393 KB)

## Data Availability

All data used in this study can be freely accessed at the GBD 2019 (http://ghdx.healthdata.org/gbd-2019).

## Abbreviations

GBD: Global burden of disease
AAPCs: Average annual percentage changes
DALYs: Disability-adjusted life-years
HBV: Hepatitis B virus
HCV: Hepatitis C virus
ALD: Alcoholic liver disease
NAFLD: Non-alcoholic fatty liver disease
SDI: Sociodemographic index
APC: Annual percent change
CI: Confidence interval
UI: Uncertainty interval
