# Supplementary material for: Global, regional, and national burdens of cirrhosis in children and adolescents aged under 19 years from 1990 to 2019
Source: Hepatol Int. 2023 Apr 26;18(1):238–53. doi: 10.1007/s12072-023-10531-y (PMC10858162; doi:10.1007/s12072-023-10531-y)
Supplement: Supplementary file 1 — Supplementary file1 (PDF 10393 KB) [file 12072_2023_10531_MOESM1_ESM.pdf]

Table S1 Global APCs in incidence, prevalence, mortality, and DALYs.

Time period	Incidence			Prevalence			Mortality			DALYs	
	APC (95% CI)	P-Value		APC (95% CI)	P-Value		APC (95% CI)	P-Value		APC (95% CI)	P-Value
1990 ~ 1996	0.1 (0 to 0.1)	0.028	1990 ~ 2001	-0.8 (-0.9 to -0.7)	<0.001	1990 ~ 2003	-1.3 (-1.5 to -1.2)	<0.001	1990 ~ 2003	-1.5 (-1.6 to -1.3)	<0.001
1996 ~ 2006	0.5 (0.5 to 0.5)	<0.001	2001 ~ 2006	-2.4 (-2.9 to -2)	<0.001	2003 ~ 2006	0 (-1.7 to 1.7)	0.977	2003 ~ 2006	-0.1 (-1.7 to 1.5)	0.897
2006 ~ 2009	-1 (-1.3 to -0.8)	<0.001	2006 ~ 2011	-3.1 (-3.5 to -2.6)	<0.001	2006 ~ 2019	-2.4 (-2.5 to -2.3)	<0.001	2006 ~ 2019	-2.4 (-2.4 to -2.3)	<0.001
2009 ~ 2017	-0.1 (-0.1 to -0.1)	<0.001	2011 ~ 2019	-3.7 (-3.9 to -3.5)	<0.001						
2017 ~ 2019	1.2 (0.9 to 1.5)	<0.001									

AAPC: average annual percentage changes; DALYs: disability-adjusted life-years

Table S2 Prevalence and Deaths of cirrhosis in children and adolescents at global and regional levels.

**Subgroup**	Prevalence					Deaths				
	No., 1990	No., 2019	Rate, 1990	Rate, 2019	No. change (%)	No., 1990	No., 2019	Rate, 1990	Rate, 2019	No. change (%)
**Global**	172137284 (151984780 -	101226510 (87226560 -	7571.2 (6684.8 -	3924.6 (3381.8 -	-41.2	39448 (28828 -	27577 (22588 -	1.7 (1.3	1.1 (0.9	-30.1

	194780589)	117688500)	8567.2)	4562.9)		52137)	33690)	- 2.3)	- 1.3)	
**Sex**										
Female	74093299 (65573523 - 83724159)	42935866 (37100695 - 49784726)	6682.3 (5913.9 - 7550.8)	3435.4 (2968.5 - 3983.4)	-42.1	19312 (13375 - 26522)	12302 (9956 - 15008)	1.7 (1.2 - 2.4)	1 (0.8 - 1.2)	-36.3
Male	98043985 (86700841 - 110586105)	58290644 (49904702 - 68262753)	8417.5 (7443.6 - 9494.3)	4384.5 (3753.7 - 5134.6)	-40.5	20136 (14865 - 27259)	15274 (12241 - 18926)	1.7 (1.3 - 2.3)	1.1 (0.9 - 1.4)	-24.1
**Age**										
<5 years	31275036 (26581324 - 36604782)	9799003 (8322431 - 11407751)	4947.7 (4205.1 - 5790.8)	1478.3 (1255.6 - 1721)	-68.7	15916 (9077 - 24235)	7355 (5341 - 10210)	2.5 (1.4 - 3.8)	1.1 (0.8 - 1.5)	-53.8
5-9 years	38965778 (33820325 - 44981409)	15900212 (13623096 - 18432813)	6659 (5779.6 - 7687)	2428.6 (2080.8 - 2815.4)	-59.2	7308 (5097 - 9761)	4522 (3471 - 5788)	1.2 (0.9 - 1.7)	0.7 (0.5 - 0.9)	-38.1
10-14 years	39972035 (35104321 - 45468097)	20400972 (17766273 - 23297752)	7447.9 (6540.9 - 8472)	3176.8 (2766.5 - 3627.9)	-49.0	5510 (4431 - 6728)	4802 (3921 - 5798)	1 (0.8 - 1.3)	0.7 (0.6 - 0.9)	-12.8
15-19	61924435	55126323	11917.6	8897.9	-11.0	10714	10898	2.1	1.8	1.7

years	(53721292 - 72313016)	(44792969 - 69201279)	(10338.9 - 13917)	(7230 - 11169.8)		(9271 - 12484)	(9290 - 12653)	(1.8 - 2.4)	(1.5 - 2)	
**Cause**										
alcohol use	3133 (764 - 7052)	4193 (1005 - 9544)	0.1 (0 - 0.3)	0.2 (0 - 0.4)	33.8	40 (12 - 89)	39 (11 - 88)	0 (0 - 0)	0 (0 - 0)	-2.5
hepatitis B	125053975 (107448421 - 144188353)	46400334 (39395898 - 54065235)	5500.3 (4726 - 6341.9)	1799 (1527.4 - 2096.1)	-62.9	1177 (638 - 1850)	878 (476 - 1388)	0.1 (0 - 0.1)	0 (0 - 0.1)	-25.4
hepatitis C	25097243 (19340068 - 32286028)	22573176 (16986163 - 29415788)	1103.9 (850.6 - 1420.1)	875.2 (658.6 - 1140.5)	-10.1	1147 (636 - 1880)	1027 (571 - 1659)	0.1 (0 - 0.1)	0 (0 - 0.1)	-10.5
NAFLD	19368044 (12876693 - 28650239)	29160943 (19465876 - 42848775)	851.9 (566.4 - 1260.1)	1130.6 (754.7 - 1661.3)	50.6	261 (105 - 518)	261 (105 - 523)	0 (0 - 0)	0 (0 - 0)	0.0
other causes	2614890 (2017707 - 3329832)	3087864 (2372099 - 3919409)	115 (88.7 - 146.5)	119.7 (92 - 152)	18.1	36823 (26496 - 49199)	25372 (20586 - 31245)	1.6 (1.2 - 2.2)	1 (0.8 - 1.2)	-31.1
**SDI**										
Low SDI	27429900 (23811316 - 31457629)	33637357 (29347107 - 38406484)	9286.6 (8061.5 - 10650.2)	5634.7 (4916.1 - 6433.6)	22.6	8807 (5499 - 13174)	10733 (8292 - 13692)	3 (1.9 - 4.5)	1.8 (1.4 - 2.3)	21.9
Low-	37624849	27317839	6594	3928.6	-27.4	15791	10065	2.8	1.4	-36.3

middle SDI	(33013246 - 42874244)	(23814005 - 31525266)	(5785.8 - 7514)	(3424.7 - 4533.7)		(11135 - 21354)	(8112 - 12419)	(2 - 3.7)	(1.2 - 1.8)	
Middle SDI	71531704 (63441453 - 80459692)	26892964 (22483134 - 32222014)	9327.1 (8272.2 - 10491.2)	3651.2 (3052.5 - 4374.7)	-62.4	11471 (8842 - 14643)	5558 (4635 - 6691)	1.5 (1.2 - 1.9)	0.8 (0.6 - 0.9)	-51.5
High-middle SDI	28819570 (25630109 - 32397939)	8888926 (7102825 - 11070319)	7111.6 (6324.5 - 7994.6)	2710.3 (2165.7 - 3375.4)	-69.2	2820 (2364 - 3351)	1051 (898 - 1226)	0.7 (0.6 - 0.8)	0.3 (0.3 - 0.4)	-62.7
High SDI	6655715 (5768823 - 7699591)	4429118 (3494283 - 5642981)	2842.3 (2463.6 - 3288.1)	2005.7 (1582.4 - 2555.4)	-33.5	534 (492 - 572)	154 (135 - 171)	0.2 (0.2 - 0.2)	0.1 (0.1 - 0.1)	-71.2
**Region**										
Andean Latin America	336300 (276427 - 410958)	444762 (336282 - 579869)	1757.4 (1444.6 - 2147.6)	1878.2 (1420.1 - 2448.7)	32.3	557 (404 - 813)	149 (107 - 203)	2.9 (2.1 - 4.3)	0.6 (0.5 - 0.9)	-73.2
Australasia	189218 (162370 - 220290)	127747 (99128 - 161948)	3015.5 (2587.6 - 3510.7)	1772.8 (1375.7 - 2247.4)	-32.5	4 (3 - 5)	2 (2 - 3)	0.1 (0.1 - 0.1)	0 (0 - 0)	-50.0
Caribbean	439464 (364675	441564	2912.6	2842.9	0.5	306 (176	158 (85 -	2	1	-48.4

	- 521455)	(356869 - 538496)	(2416.9 - 3456)	(2297.6 - 3467)		- 499)	258)	(1.2 - 3.3)	(0.5 - 1.7)	
Central Asia	2508860 (2095898 - 2881528)	1501374 (1234096 - 1776826)	7954.1 (6644.8 - 9135.6)	4418.1 (3631.6 - 5228.6)	-40.2	611 (547 - 685)	462 (399 - 533)	1.9 (1.7 - 2.2)	1.4 (1.2 - 1.6)	-24.4
Central Europe	1000665 (849320 - 1171232)	417157 (325092 - 532302)	2595.1 (2202.6 - 3037.4)	1783.1 (1389.5 - 2275.2)	-58.3	202 (183 - 237)	37 (32 - 43)	0.5 (0.5 - 0.6)	0.2 (0.1 - 0.2)	-81.7
Central Latin America	2446313 (2019243 - 2902290)	2240386 (1722534 - 2866118)	2972.7 (2453.7 - 3526.8)	2561.9 (1969.8 - 3277.5)	-8.4	1180 (1060 - 1328)	468 (386 - 568)	1.4 (1.3 - 1.6)	0.5 (0.4 - 0.6)	-60.3
Central Sub-Saharan Africa	3478956 (2962482 - 4042880)	5081237 (4385824 - 5822067)	11038.2 (9399.5 - 12827.5)	7140.5 (6163.2 - 8181.5)	46.1	851 (580 - 1239)	1109 (611 - 1996)	2.7 (1.8 - 3.9)	1.6 (0.9 - 2.8)	30.3
East Asia	63886870 (56573241 - 71729208)	9940670 (8239035 - 12022465)	13725.4 (12154.1 - 15410.2)	3197.5 (2650.2 - 3867.1)	-84.4	2468 (1915 - 2978)	324 (270 - 389)	0.5 (0.4 - 0.6)	0.1 (0.1 - 0.1)	-86.9
Eastern Europe	1997354 (1697477 -	1089092 (858309 -	2967.2 (2521.7 -	2296.5 (1809.9 -	-45.5	239 (222 - 270)	108 (93 - 123)	0.4 (0.3	0.2 (0.2	-54.8

	2317407)	1342804)	3442.6)	2831.5)				- 0.4)	- 0.3)	
Eastern Sub-Saharan Africa	9610382 (8257149 - 11115212)	9148522 (7958934 - 10427257)	8686.8 (7463.6 - 10047)	4092.9 (3560.7 - 4665)	-4.8	2742 (1644 - 3953)	3160 (2177 - 4396)	2.5 (1.5 - 3.6)	1.4 (1 - 2)	15.2
High-income Asia Pacific	1912201 (1652619 - 2202800)	788503 (656528 - 945767)	3795.2 (3280 - 4372)	2441.4 (2032.8 - 2928.4)	-58.8	198 (170 - 218)	19 (16 - 22)	0.4 (0.3 - 0.4)	0.1 (0 - 0.1)	-90.4
High-income North America	1340664 (1076536 - 1668205)	1508244 (1111011 - 2001217)	1646 (1321.7 - 2048.1)	1676.9 (1235.3 - 2225.1)	12.5	132 (120 - 141)	65 (59 - 71)	0.2 (0.1 - 0.2)	0.1 (0.1 - 0.1)	-50.8
Mexico	894011 (690150 - 1129356)	999089 (727927 - 1307724)	2057.9 (1588.7 - 2599.7)	2311.8 (1684.3 - 3025.9)	11.8	519 (427 - 616)	209 (178 - 242)	1.2 (1 - 1.4)	0.5 (0.4 - 0.6)	-59.7
North Africa and Middle East	13489409 (11746396 - 15459074)	11016532 (8859396 - 13842265)	7481.9 (6515.1 - 8574.3)	4815 (3872.2 - 6050.1)	-18.3	3698 (2150 - 5408)	2230 (1746 - 2839)	2.1 (1.2 - 3)	1 (0.8 - 1.2)	-39.7
Oceania	315382 (248398 - 365408)	286220 (246164 - 326673)	9527.5 (7504 - 11038.8)	4685.7 (4029.9 - 5348)	-9.2	31 (22 - 40)	44 (31 - 63)	0.9 (0.7 -	0.7 (0.5 - 1)	41.9

								1.2)		
South Asia	24007310 (20818347 - 27711286)	22349077 (19233628 - 25975659)	4380.4 (3798.6 - 5056.2)	3217.9 (2769.3 - 3740.1)	-6.9	15690 (10563 - 21415)	10520 (8285 - 13090)	2.9 (1.9 - 3.9)	1.5 (1.2 - 1.9)	-33.0
Southeast Asia	18903939 (16556642 - 21438324)	8956128 (7630214 - 10505408)	8542.8 (7482 - 9688)	3972.7 (3384.5 - 4659.9)	-52.6	5769 (3588 - 8409)	2680 (2285 - 3139)	2.6 (1.6 - 3.8)	1.2 (1 - 1.4)	-53.5
Southern Latin America	200664 (160129 - 253923)	219908 (161565 - 295926)	1035.8 (826.6 - 1310.7)	1101.2 (809.1 - 1481.9)	9.6	90 (81 - 100)	42 (35 - 50)	0.5 (0.4 - 0.5)	0.2 (0.2 - 0.2)	-53.3
Southern Sub-Saharan Africa	2802947 (2516906 - 3102172)	1409232 (1230752 - 1614811)	10695 (9603.6 - 11836.8)	4603.3 (4020.3 - 5274.8)	-49.7	235 (179 - 301)	141 (99 - 194)	0.9 (0.7 - 1.1)	0.5 (0.3 - 0.6)	-40.0
Tropical Latin America	3918530 (3373739 - 4547775)	1570233 (1229739 - 1952721)	5627.4 (4845 - 6531)	2350.2 (1840.5 - 2922.6)	-59.9	749 (660 - 866)	221 (194 - 251)	1.1 (0.9 - 1.2)	0.3 (0.3 - 0.4)	-70.5
Western Europe	1900600 (1561879 - 2328360)	1443361 (1108577 - 1867597)	1931.4 (1587.2 - 2366.1)	1564.6 (1201.7 - 2024.5)	-24.1	158 (146 - 176)	43 (39 - 50)	0.2 (0.1 - 0.2)	0 (0 - 0.1)	-72.8

Western Sub-Saharan Africa	17451258 (15323718 - 19707218)	21246562 (18635184 - 24082854)	16257.8 (14275.7 - 18359.4)	8557.7 (7505.9 - 9700.1)	21.7	3538 (2487 - 5159)	5593 (4050 - 7428)	3.3 (2.3 - 4.8)	2.3 (1.6 - 3)	58.1

SDI: socio-demographic index

Table S3 Subgroups AAPCs in prevalence and deaths.

	Prevalence		Deaths	
	AAPC (95% CI)	P-Value	AAPC (95% CI)	P-Value
**Sex**				
Female	-2.3 (-2.4 to -2.1)	<0.001	-2 (-2.1 to -1.8)	<0.001
Male	-2.2 (-2.4 to -2.1)	<0.001	-1.4 (-1.6 to -1.1)	<0.001
**Age**				
<5 years	-4.1 (-4.3 to -3.9)	<0.001	-2.8 (-2.9 to -2.7)	<0.001
5-9 years	-3.4 (-3.6 to -3.3)	<0.001	-2.2 (-2.3 to -2)	<0.001
10-14 years	-2.9 (-3.1 to -2.6)	<0.001	-1.1 (-1.3 to -1)	<0.001
15-19 years	-1 (-1.1 to -1)	<0.001	-0.7 (-0.8 to -0.6)	<0.001
**Cause**				
alcohol use	0.6 (0.4 to 0.8)	<0.001	-0.4 (-0.9 to 0)	0.048
hepatitis B	-3.8 (-3.9 to -3.6)	<0.001	-1.4 (-1.7 to -1.1)	<0.001
hepatitis C	-0.8 (-0.9 to -0.7)	<0.001	-0.8 (-1 to -0.6)	<0.001
NAFLD	1 (0.9 to 1.1)	<0.001	-0.4 (-0.8 to -0.1)	0.019
other causes	0.1 (0.1 to 0.2)	<0.001	-1.7 (-1.9 to -1.6)	<0.001
**SDI**				
Low SDI	-1.7 (-1.9 to -1.6)	<0.001	-1.7 (-1.9 to -1.5)	<0.001

Low-middle SDI	-1.8 (-1.9 to -1.7)	<0.001	-2.2 (-2.4 to -2)	<0.001
Middle SDI	-3.2 (-3.3 to -3.1)	<0.001	-2.3 (-2.6 to -2)	<0.001
High-middle SDI	-3.3 (-3.5 to -3)	<0.001	-2.6 (-3 to -2.2)	<0.001
High SDI	-1.2 (-1.3 to -1.1)	<0.001	-3.9 (-4.1 to -3.7)	<0.001
**Region**				
Andean Latin America	0.2 (0.2 to 0.3)	<0.001	-5.1 (-5.5 to -4.8)	<0.001
Australasia	-1.8 (-2 to -1.6)	<0.001	-2.8 (-3.5 to -2.1)	<0.001
Caribbean	-0.1 (-0.2 to 0)	0.007	-2.4 (-2.6 to -2.1)	<0.001
Central Asia	-2 (-2.1 to -1.9)	<0.001	-1.2 (-1.5 to -0.9)	<0.001
Central Europe	-1.3 (-1.4 to -1.2)	<0.001	-3.9 (-4.4 to -3.4)	<0.001
Central Latin America	-0.5 (-0.6 to -0.4)	<0.001	-3.3 (-3.5 to -3.1)	<0.001
Central Sub-Saharan Africa	-1.5 (-1.6 to -1.4)	<0.001	-1.9 (-2.1 to -1.7)	<0.001
East Asia	-4.9 (-5.1 to -4.6)	<0.001	-5.6 (-5.9 to -5.3)	<0.001
Eastern Europe	-0.9 (-1 to -0.8)	<0.001	-1.6 (-2.7 to -0.5)	0.003
Eastern Sub-Saharan Africa	-2.6 (-2.8 to -2.4)	<0.001	-2 (-2.1 to -1.9)	<0.001
High-income Asia Pacific	-1.6 (-1.7 to -1.4)	<0.001	-6.3 (-6.6 to -5.9)	<0.001
High-income North America	0.1 (0 to 0.2)	0.022	-2.7 (-3.1 to -2.3)	<0.001
Mexico	0.4 (0.4 to 0.4)	<0.001	-2.9 (-3.3 to -2.6)	<0.001
North Africa and Middle East	-1.5 (-1.6 to -1.4)	<0.001	-2.5 (-2.7 to -2.3)	<0.001
Oceania	-2.4 (-2.5 to -2.3)	<0.001	-0.9 (-1.2 to -0.5)	<0.001
South Asia	-1.1 (-1.1 to -1)	<0.001	-2.1 (-2.6 to -1.6)	<0.001
Southeast Asia	-2.6 (-2.8 to -2.5)	<0.001	-2.6 (-2.8 to -2.5)	<0.001
Southern Latin America	0.2 (0.1 to 0.3)	<0.001	-2.6 (-3.2 to -2.1)	<0.001
Southern Sub-Saharan Africa	-2.8 (-3.1 to -2.5)	<0.001	-2.2 (-2.9 to -1.6)	<0.001
Tropical Latin America	-3 (-3.3 to -2.7)	<0.001	-4 (-4.3 to -3.6)	<0.001

Western Europe	-0.7 (-0.8 to -0.7)	<0.001	-4.2 (-4.5 to -4)	<0.001
Western Sub-Saharan Africa	-2.2 (-2.3 to -2)	<0.001	-1.3 (-1.5 to -1.1)	<0.001

SDI: socio-demographic index

Figure S1 Prevalence and deaths of CLD in subgroups in children and adolescents from 1990 to 2019.
(A) Sex subgroup; (B) Age subgroup; (C) Causes subgroup.

Figure S2 The percentage change of CLD between 1990 and 2019. (A) Incidence; (B) Prevalence; (C) Deaths; (D) DALYs.
DALYs: disability-adjusted life-years

Figure S3 Geographical distribution of CLD prevalence in children and adolescents in 204 countries and territories.
(A) Prevalence of cirrhosis in 2019; (B) AAPC in prevalence of cirrhosis between 1990 and 2019.
AAPC: average annual percentage change

Figure S4 Geographical distribution of CLD deaths in children and adolescents in 204 countries and territories.
(A) Deaths of cirrhosis in 2019; (B) AAPC in deaths rate of cirrhosis between 1990 and 2019.
AAPC: average annual percentage change

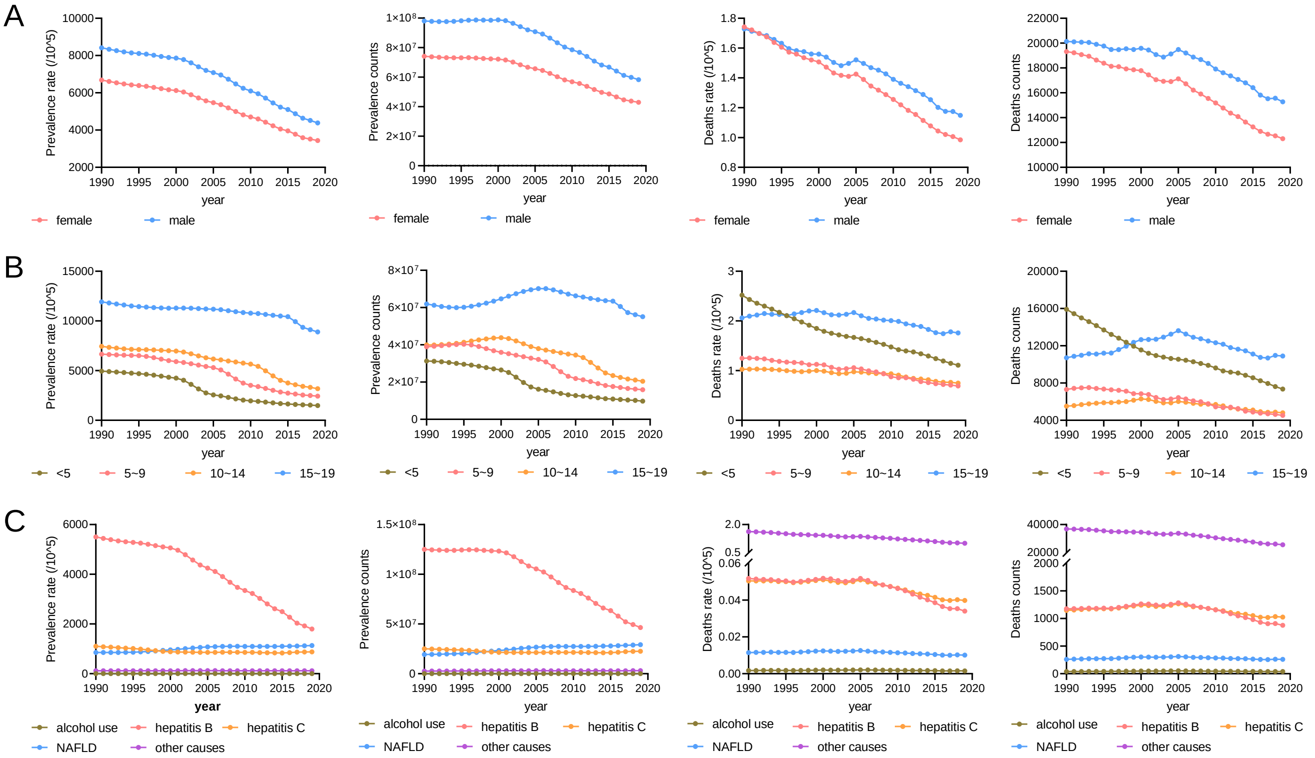
A
B
C
Prevalence rate (/10^5)
Prevalence counts
Deaths rate (/10^5)
Deaths counts
year
female
male
<5
5~9
10~14
15~19
alcohol use
hepatitis B
hepatitis C
NAFLD
other causes

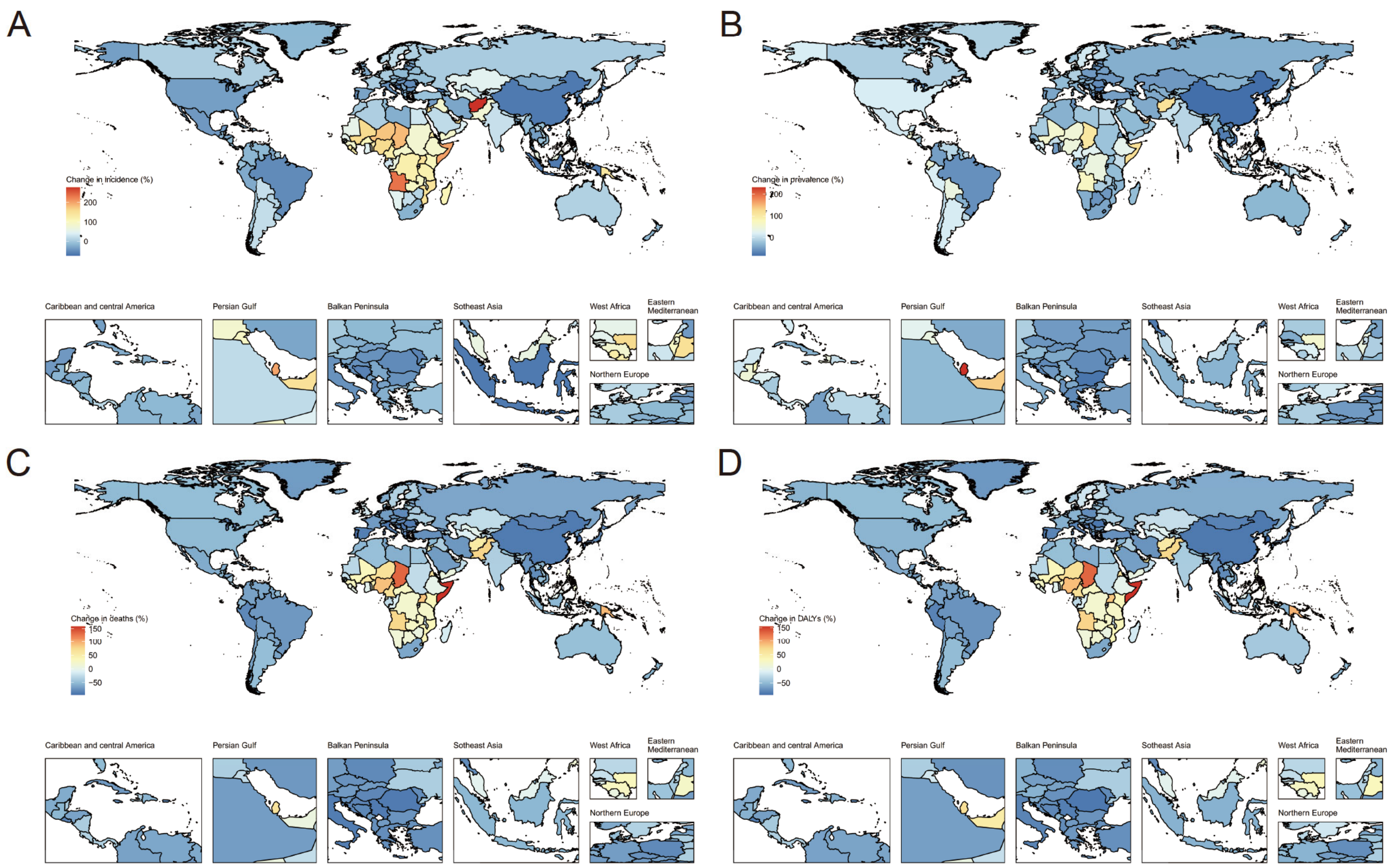
A
Change in incidence (%)
200
100
0
Caribbean and central America
Persian Gulf
Balkan Peninsula
Sotheast Asia
West Africa
Eastern Mediterranean
Northern Europe
B
Change in prevalence (%)
200
100
0
Caribbean and central America
Persian Gulf
Balkan Peninsula
Sotheast Asia
West Africa
Eastern Mediterranean
Northern Europe
C
Change in deaths (%)
150
100
50
0
−50
Caribbean and central America
Persian Gulf
Balkan Peninsula
Sotheast Asia
West Africa
Eastern Mediterranean
Northern Europe
D
Change in DALYs (%)
150
100
50
0
−50
Caribbean and central America
Persian Gulf
Balkan Peninsula
Sotheast Asia
West Africa
Eastern Mediterranean
Northern Europe

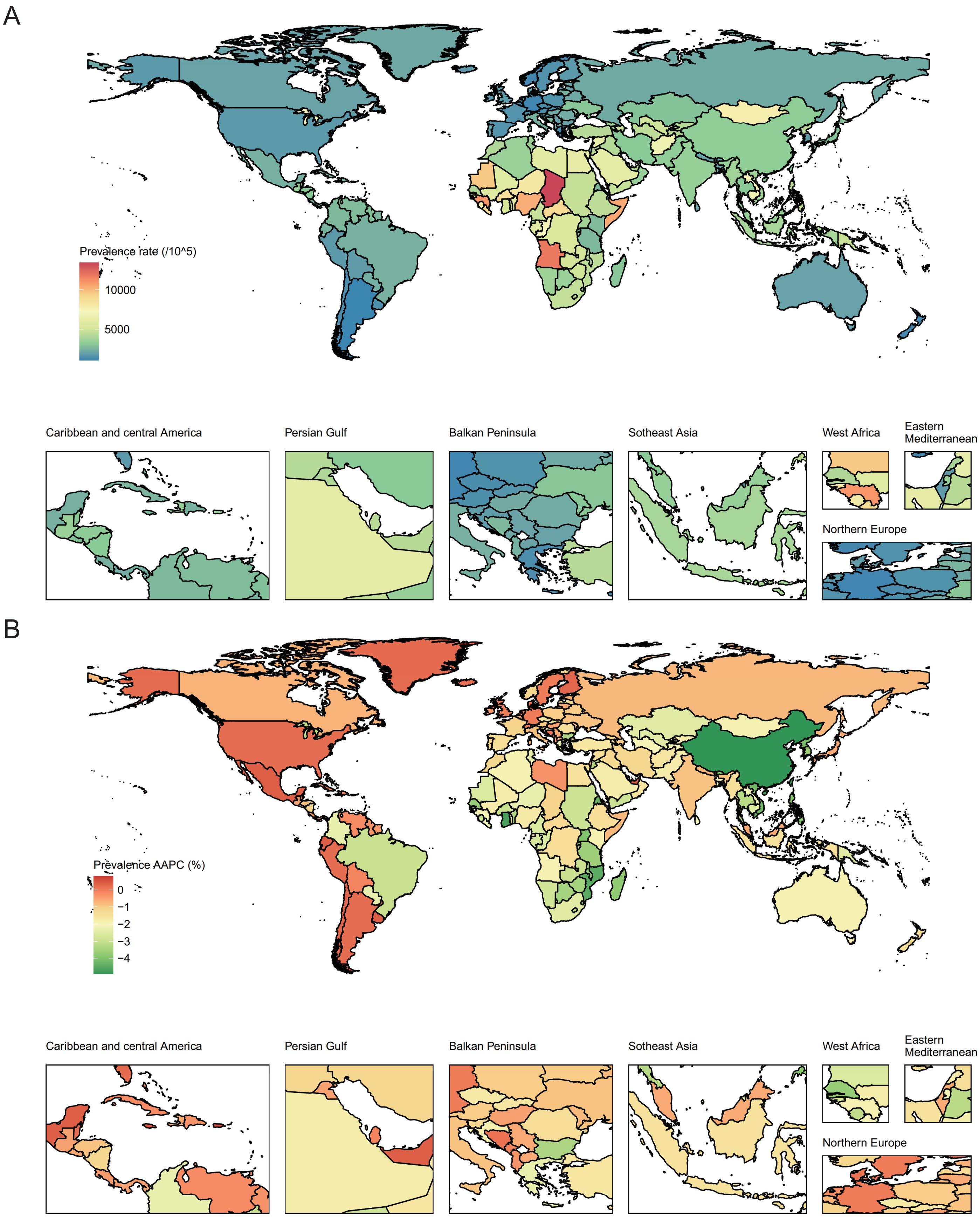

A
Prevalence rate (/10^5)
10000
5000
Caribbean and central America
Persian Gulf
Balkan Peninsula
Sotheast Asia
West Africa
Eastern Mediterranean
Northern Europe
B
Prevalence AAPC (%)
0
−1
−2
−3
−4
Caribbean and central America
Persian Gulf
Balkan Peninsula
Sotheast Asia
West Africa
Eastern Mediterranean
Northern Europe

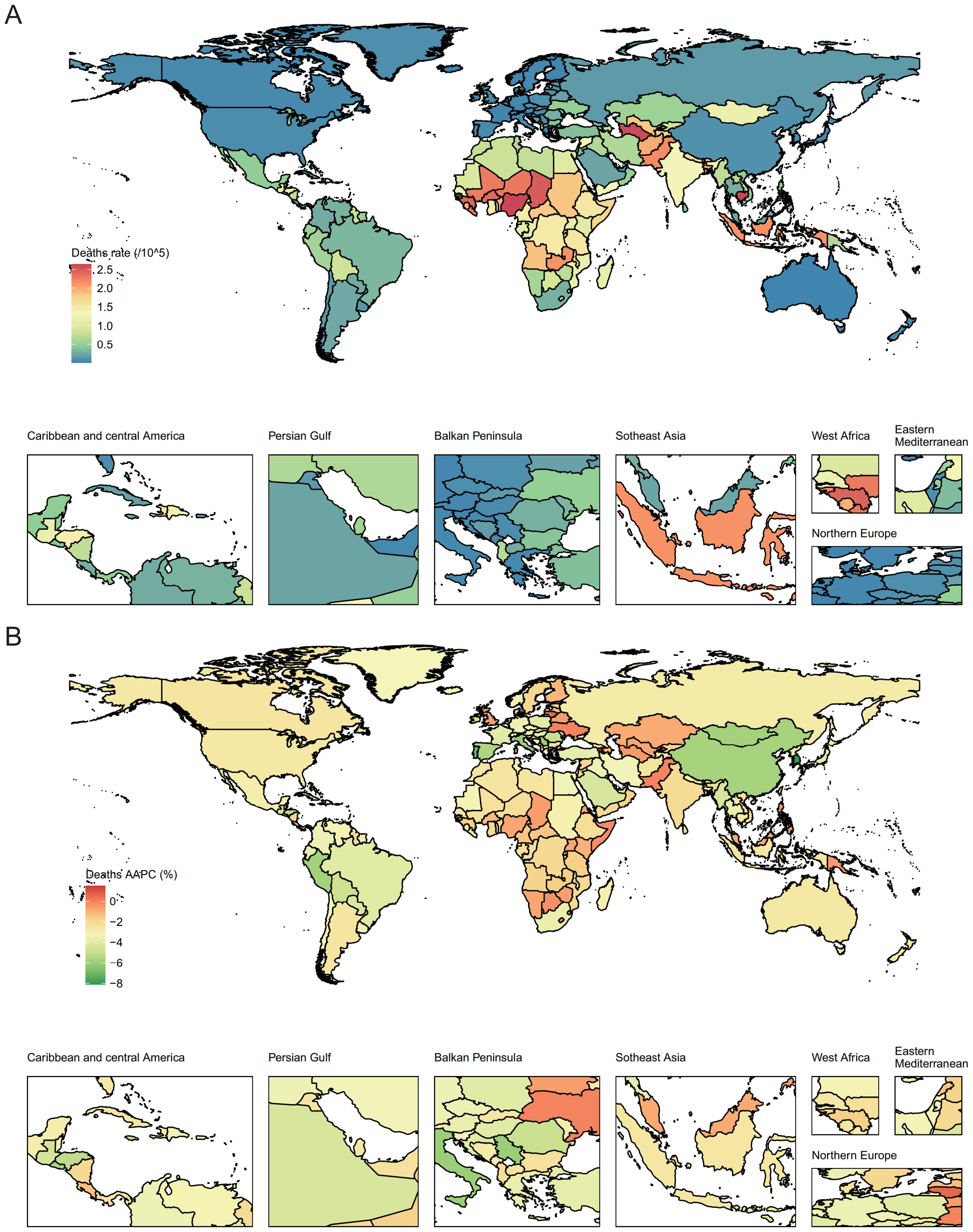
A
Deaths rate (/10^5)
2.5
2.0
1.5
1.0
0.5
Caribbean and central America
Persian Gulf
Balkan Peninsula
Sotheast Asia
West Africa
Eastern Mediterranean
Northern Europe
B
Deaths AAPC (%)
0
−2
−4
−6
−8
Caribbean and central America
Persian Gulf
Balkan Peninsula
Sotheast Asia
West Africa
Eastern Mediterranean
Northern Europe
